# The *OsERF115/AP2EREBP110* Transcription Factor Is Involved in the Multiple Stress Tolerance to Heat and Drought in Rice Plants

**DOI:** 10.3390/ijms22137181

**Published:** 2021-07-02

**Authors:** Seong-Im Park, Hyeok Jin Kwon, Mi Hyeon Cho, Ji Sun Song, Beom-Gi Kim, JeongHo Baek, Song Lim Kim, HyeonSo Ji, Taek-Ryoun Kwon, Kyung-Hwan Kim, In Sun Yoon

**Affiliations:** 1 Gene Engineering Division, National Institute of Agricultural Sciences, RDA, Jeonju 54874, Korea; sipark528@korea.kr (S.-I.P.); littlekhj@naver.com (H.J.K.); chomi1@korea.kr (M.H.C.); songmari@korea.kr (J.S.S.); firstleon@korea.kr (J.B.); greenksl5405@korea.kr (S.L.K.); jhs77@korea.kr (H.J.); trkwon@korea.kr (T.-R.K.); biopiakim@korea.kr (K.-H.K.); 2 Metabolic Engineering Division, National Institute of Agricultural Sciences, RDA, Jeonju 54874, Korea; bgkimpeace@korea.kr

**Keywords:** rice, OsERF115/AP2EREBP110, heat and drought tolerance, water use efficiency

## Abstract

The AP2/EREBP family transcription factors play important roles in a wide range of stress tolerance and hormone signaling. In this study, a heat-inducible rice *ERF* gene was isolated and functionally characterized. The *OsERF115/AP2EREBP110* was categorized to Group-IIIc of the rice AP2/EREBP family and strongly induced by heat and drought treatment. The OsERF115/AP2EREBP110 protein targeted to nuclei and suppressed the ABA-induced transcriptional activation of *Rab16A* promoter in rice protoplasts. Overexpression of *OsERF115/AP2EREBP110* enhanced thermotolerance of seeds and vegetative growth stage plants. The *OsERF115/AP2EREBP110* overexpressing (OE) plants exhibited higher proline level and increased expression of a proline biosynthesis *P5CS1* gene. Phenotyping of water use dynamics of the individual plant indicates that the *OsERF115/AP2EREBP110*-OE plant exhibited better water saving traits under heat and drought combined stress. Our combined results suggest the potential use of *OsERF115/AP2EREBP110* as a candidate gene for genetic engineering approaches to develop heat and drought stress-tolerant crops.

## 1. Introduction

Global food security is being challenged by the rapid growth of the world’s population and extreme climate change [1]. High temperature and drought, representative environmental stresses caused by climate change, have become major limiting factors affecting crop productivity and ultimately food security [2]. The reduced precipitation and changes in precipitation patterns are causing frequent droughts worldwide [3]. In addition, the increase in annual average temperature due to increased concentration of greenhouse gases has led to the simultaneous occurrence of heat and drought stressors on arable land worldwide [4]. These studies predict that extreme drought conditions and increasing temperature is expected to worsen in the coming decades.

Both high temperature and drought increase plant leaf temperature, significantly reducing the water use efficiency, relative water content, transpiration rate, and photosynthetic activity [5,6,7]. Consequently, these stresses reduce crop yield by inhibiting plant growth and increasing infertility during the vegetation and reproduction growth stages of the plant [8,9,10,11]. Therefore, it is important to study the plant response mechanism to heat and drought stress and to develop stress-tolerant plants using genetic resources. Although many studies on heat and drought stress responses have been conducted over the years, the majority of these studies are about the effects of a single stress on plants [12,13,14,15,16]. In fact, several studies have shown that the combined stress of heat and drought has caused more damage to plant growth and productivity compared to a single stress factor [2,7,17]. Combined stress can also alter plant metabolism in novel ways that may be different from those triggered by an individual stress [18]. For this reason, future research should focus on the mechanisms of response to complex stress combined with heat and drought.

In recent years, many new promising plant phenotyping techniques have been implemented to study agronomical traits related to stress response and tolerance in plants [19]. Phenotypic analysis based on the non-destructive and automated high-throughput red-blue-green (RGB) imaging, chlorophyll fluorescence imaging, infrared (IR) thermal imaging, near infrared (NIR) imaging, and hyperspectral imaging techniques has been widely applied to assess drought, salinity, or the heat tolerance of diverse crops and Arabidopsis [20,21,22,23,24,25,26]. In particular, heat and drought stress tolerance, primarily determined by efficient water use and ability to prevent water loss, could be assessed through IR thermography among phenotyping tools. Since water content and transpiration state of leaf can be indirectly estimated by leaf temperature changes [27], IR thermal imaging is used to accurately and quickly determine the plant temperature change [28]. Therefore, this method has been recently applied to study the response to heat and drought stress [29,30,31,32,33,34].

Plants have evolved complex and elaborate adaptation mechanisms to cope with these environmental stresses. These mechanisms involve morphological, physiological, and biochemical changes by regulating the expression of stress-responsive genes through stress-specific signaling pathways. Transcription factors are essential for regulating gene expression by interacting with *cis*-acting elements in the promoter regions of stress-related genes in stress signaling and adaptive networks [35]. Studies on the overexpression of transcription factor genes such as *NAC*, *MYB*, *bZIP*, and *AP2/ERF* suggest that transcriptional modulation plays an important role in the abiotic stress response of plants [36].

The plant-specific AP2/EREBP (APETALA2/Ethylene Responsive Element Binding Protein) transcription factors are a large group with 170 genes in rice that regulate key functions of response to several abiotic stresses and hormones [37]. The AP2/EREBP family is characterized by the one or two conserved AP2/ERF domain, which comprises 60–70 amino acid residues [38]. This family contains four major subfamilies: AP2, RELATED TO ABSCISIC ACID INSENSITIVE3/VIVIPAROUS 1 (RAV), ERFs, and DEHYDRATION-RESPONSIVE ELEMENT BINDING proteins (DREBs) [39]. The ERF subfamily specifically binds to the core *cis*-acting element, AGCCGCC (GCC box). Expression of the ERF subfamily genes have been reported to be induced by multiple stresses in various species and function as a hub protein in the stress and hormone-responsive signaling pathway [40,41,42].

We have identified the *OsERF115/AP2EREBP110* gene as a heat-inducible transcription factor from the microarray analysis of ripening rice seeds. Based on the phylogenetic tree of the ERF gene family, the *OsERF115/AP2EREBP110* belongs to a small distinct subgroup X [38] or subgroup IIIc [37], whose function has been mostly unidentified yet. One previous report showed that the OsERF115 interacts with an aleurone layer specific OsNF-YB1 protein associated with grain filling and endosperm development [43]. However, it is unknown what the function of *OsERF115/AP2EREBP110* is in abiotic stress responses. In the present study, *OsERF115/AP2EREBP110*-OE transgenic rice plants were constructed and their function in heat and drought stress responses was investigated. Phenotyping of seeds and seedlings for thermotolerance and water use dynamics revealed that overexpression of *OsERF115/AP2EREBP110* enhanced tolerance under the heat-drought combined stress and improved water saving traits of rice. Our results suggest that *OsERF115/AP2EREBP110* is a promising candidate for the transgenic breeding of plants with enhanced tolerance to both heat and drought stress.

## 2. Results

### 2.1. OsERF115/AP2EREBP110 Was Identified as a Heat-Inducible Transcription Factor by Microarray Analysis of Ripening Rice Seeds

**Figure 1:**
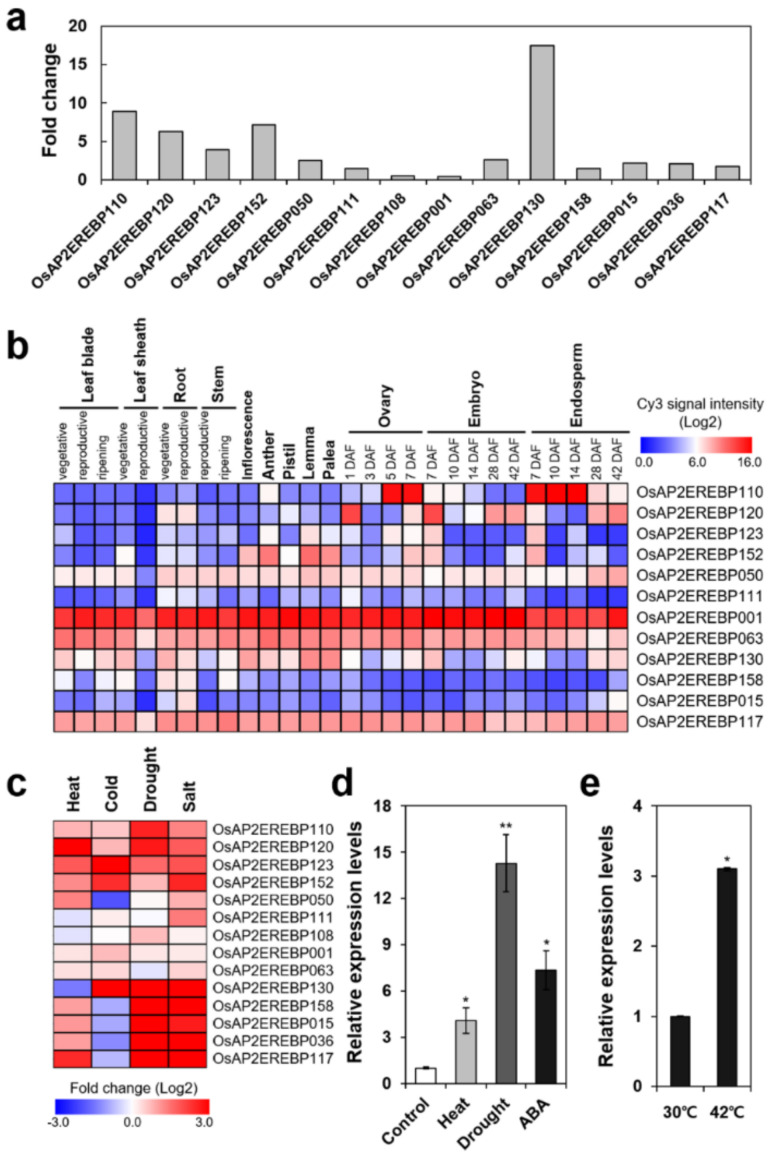
Expression profile of *OsAP2/EREBP* Group-IIIc genes. (**a**) Heat stress-induced transcript level of *OsAP2/EREBP* Group-IIIc genes in ripening seeds. Rice panicles at 25 DAH were incubated for 5 days at 30 °C and 42 °C, respectively. Transcriptomic profiling was analyzed using the Agilent *O. sativa* GE 180K microarray platform. Data presented as fold change of normalized intensity values for 16 Group-IIIc genes from heat treated seeds compared to the control. (**b**) Spatio-temporal gene expression analysis of *OsAP2/EREBP* Group-IIIc members in various tissues/organs at different developmental stages. Heatmap of the normalized Cy3 signal intensity values for 12 *OsAP2/EREBP* Group-IIIc genes was constructed according to the RiceXPro database. (**c**) Expression analysis of *OsAP2/EREBP* Group-IIIc genes in rice seedlings under various abiotic stresses. Heatmap of the normalized intensity values for 14 Group-IIIc was constructed according to the public microarray data GSE14275 and GSE6901. (**d**) qRT-PCR analysis of *OsERF115/AP2EREBP110* (*LOC_Os08g41030*) gene in rice seedlings subjected to heat (42 °C), drought, or 3 μM ABA treatment. Data represent mean ± standard deviation (SD) from three independent experiments with three biological replicates (*n* = 3). One-way ANOVA and Tukey’ HSD tests were performed compared with the untreated control (*, *p* < 0.05; **, *p* < 0.01). (**e**) qRT-PCR analysis of the *OsERF115/AP2EREBP110* gene in 34 DAH seeds exposed to 30 °C or 42 °C for 5 days. Relative transcript level was determined by qRT-PCR and normalized to tubulin as an internal control. Data represent mean (± SD) from three independent experiments with three biological replicates (*n* = 3). Student’s *t*-test was performed in comparison with the 30 °C-treated seeds as the control (*, *p* < 0.05).

We analyzed heat stress-responsive gene expression profiling of ripening rice seed at 25 days after heading (DAH) by microarray analysis. When we monitored transcript levels of OsAP2/EREBP family genes, it was noted that gene expression of many Group-IIIc members was increased by heat stress in rice seeds (Figure 1a). The rice AP2/EREBP family is a large group with 170 genes. Rashid et al. categorized the rice AP2/EREBP members into 11 groups based on the motif analysis [37]. Among them, the function of Group-IIIc members was mostly unidentified. The domain structure and conserved motives of Group-IIIc are shown in Figure S1. In particular, the *OsERF115*/*AP2EREBP110* (*LOC_Os08g41030*), *OsERF98*/*AP2EREBP120* (*LOC_Os02g34260*), *OsERF104*/*AP2EREBP152* (*LOC_Os08g36920*), and *OsERF103/AP2EREBP130* (*LOC_Os02g52670*) genes were upregulated more than five-fold by heat stress in ripening seeds (Figure 1a). Analysis of spatiotemporal gene expression patterns using the public database RiceXPro2 showed that expressions of *OsAP2EREBP110*, *OsAP2EREBP120*, and *OsAP2EREBP152* are highly specific to reproductive tissues such as floral organs and seeds (Figure 1b).

We further investigated gene expression patterns of Group-IIIc subfamily members in response to diverse abiotic stress in vegetative seedlings using the public microarray database, GSE14275 and GSE6901. Among the Group-IIIc members, transcript levels of eight genes were increased more than two-fold by heat, drought, and salt stresses (Figure 1c), indicating that these Group-IIIc *OsAP2/EREBP* genes are highly responsive to abiotic stresses in vegetative tissues. The AP2/ERF transcription factors have been implicated as major regulators of chilling stress response of japonica rice [44]. Among the Group-IIIc members, expressions of *OsAP2EREBP123* (*LOC_Os09g28440*), *OsAP2EREBP130*, and *OsAP2EREBP152* genes were induced by cold stress more than two-fold (Figure 1c). In addition, the *OsERF115/AP2EREBP110* gene was previously reported as a transcription factor highly induced in early chilling response [44].

As the gene expression of *OsERF115/AP2EREBP110* showed the most reproductive organ specificity and responded to diverse abiotic stresses, we further analyzed the function of *OsERF115/AP2EREBP110* in this study. To confirm the gene expression of *OsERF115/AP2EREBP110* in vegetative tissues in response to abiotic stresses, rice seedlings were exposed to heat (42 °C), drought, or ABA, and transcript levels of *OsERF115/AP2EREBP110* were measured by qRT-PCR analysis. In accordance with the public data, *OsERF115/AP2EREBP110* was induced by heat and drought stress as well as by ABA (Figure 1d). Our qRT-PCR data also verified the heat-inducible gene expression of *OsAP2/EREBP110* in ripening seeds (Figure 1e).

### 2.2. OsERF115/AP2EREBP110 Is a Nuclear Localized Transcription Factor That Transcriptionally Represses Rab16A Promoter in Response to ABA in Rice Protoplasts

**Figure 2:**
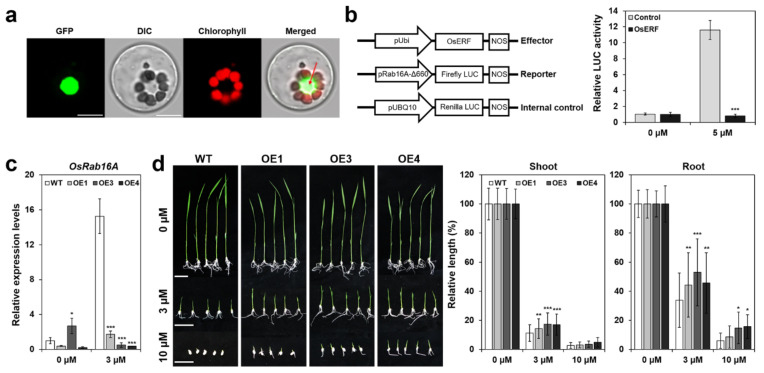
OsERF115/AP2EREBP110 is a nuclei targeted transcriptional regulator that suppress ABA-mediated transcriptional activation of *Rab16A* gene. (**a**) Subcellular localization of OsERF115/AP2EREBP110-GFP fusion protein in rice protoplasts. The rice protoplasts were transfected with *pUbi:OsERF115/AP2EREBP110*-GFP vector, incubated for 18 h, and observed by laser-scanning confocal microscopy (Leica TCS SP8). Scale bar: 10 μm (**b**) OsERF115/AP2EREBP110 suppressed ABA-induced transactivation of *Rab16A-Δ660* promoter in rice protoplasts. Rice protoplasts were co-transfected with effector (*pUbi:OsERF115/AP2EREBP110-HA*), reporter (*pRab16A-Δ660:fLUC*), and internal control (*pUbi:rLUC*) plasmids and incubated for 18 h in the presence or absence of 5 μM ABA. fLUC activity was normalized with rLUC activity. Schematic diagrams of the vector constructs were shown in the left panel. Control; *pRab16A-Δ660:fLUC* only. Data represent mean (± SD) from three independent experiments with three biological replicates. Two-way ANOVA and Fisher’s LSD test were performed compared with the control (***, *p* < 0.001). (**c**) ABA-induced expression of the *Rab16A* gene is suppressed in *OsERF115/AP2EREBP110*-OE transgenic rice. Transcript level of *Rab16A* was analyzed by qRT-PCR. Data represent mean (± SD) from three independent experiments with three biological replicates (*n* = 3). Two-way ANOVA and Fisher’s LSD tests were performed through comparison with WT plants as controls (*, *p* < 0.05; ***, *p* < 0.001). (**d**) *OsERF115/AP2EREBP110*-OE transgenic rice was less sensitive to ABA. Transgenic and WT seeds were planted on 1/2 MS medium containing 3 or 10 µM ABA for 7 days and shoot and root length were measured. Scale bars: 3 cm. Data represent mean (± SD) from two independent experiments (*n* = 25 or 50). Two-way ANOVA and Fisher’s LSD test were performed by comparison with the WT plants as controls (*, *p* < 0.05; **, *p* < 0.01; ***, *p* < 0.001).

ERFs are well-known transcription factors that regulate multiple stress responses and recognize the GCC box (GCCGCC) in the promoters of downstream defense- and stress-responsive genes [45]. In the present study, we investigated subcellular localization of the OsERF115/AP2EREBP110 protein by a transient expression assay in rice protoplasts. As shown in Figure 2a, subcellular localization of the GFP-tagged *OsERF115/AP2EREBP110* protein was strictly restrained to the nuclei of the protoplasts. ABA or GA treatment did not redistribute the nuclear-localized GFP signals (data not shown). To analyze the function of *OsERF115/AP2EREBP110* in the transcriptional regulation, the *OsERF115/AP2EREBP110* protein was fused to the GAL4 binding domain (BD) of pGBKT7 and monitored transcriptional activity in yeast cells. There was no transcriptional activation activity in yeast cells harboring the Gal4BD-OsERF115/AP2EREBP110 protein (data not shown). Our results are consistent with the previous study that *OsERF115* did not possess transactivational activity in yeast cells [43].

We further analyzed the transcriptional regulatory role of *OsERF115/AP2EREBP110* using a promoter of an ABA- and stress-responsive rice *Rab16A* gene. The rice Rab16A belongs to the LEA protein family and was highly expressed during seed maturation and in seedlings in response to diverse abiotic stress signals or ABA [46]. Overexpression of *Rab16A* displayed tolerance to salinity stress of a salt-sensitive rice cultivar and tobacco [47]. The promoter of the *Rab16A* gene contains two distinctive abscisic acid responsive elements (Motif I; ABRE) and coupling elements (Motif II; CE) [48]. Kim et al. reported that the *Rab16A* promoter fused to firefly luciferase could be used as a reporter system for ABA-dependent gene expression due to rapid and significant response to ABA in rice protoplasts [49]. In addition to ABRE and CE, the *Rab16A* promoter also possesses two drought responsive elements (DRE/CRT) and a GCC box cis element, which are known as the binding motif of DREB subfamily proteins or ERF members, respectively (Figure S3). The location of the GCC box of the *Rab16A* promoter was overlapped with the ABA responsive coupling element Motif II (Figure S3). We performed a transient transactivation assay of OsERF115/AP2EREBP110 using the *pRab16A-fLUC* reporter system in rice protoplasts. A modified construct of the *Rab16A* promoter, *pRab16A-*△*660* (same as the *pD2-2XDRE* in Min et al. [50]), which contains two DREs, one ABRE and one GCC box, was fused to the firefly luciferase gene (*fLUC*) (Figure 2b; left panel and Figure S3). As previously reported by Min et al., the *pRab16A-*△*660* was clearly activated by ABA in rice protoplasts (Figure 2b; right panel). In contrast, when *OsERF115/AP2EREBP110* was co-transfected with *pRab16A-*△*660*, the ABA-inducible fLUC activity was drastically decreased in the presence of 5 µM ABA (Figure 2b; right panel). This implicates that *OsERF115/AP2EREBP110* may act as a suppressor of ABA-induced transcriptional activation.

### 2.3. Generation of OsERF115/AP2EREBP110-OE Transgenic Rice Plants

Our results indicate that *OsERF115/AP2EREBP110* is a heat-responsive gene in seeds and vegetative seedlings (Figure 1) and exhibits a particularly high gene expression level in response to drought stress (Figure 1c,d). To further investigate the function of *OsERF115/AP2EREBP110* in rice plants associated with heat and drought stress responses, we constructed plant expression vector and transformed wild type (WT), Dongjin rice for overexpression of *OsERF115/AP2EREBP110*. As described in the Materials and Methods, a synthetic codon-optimized ORF of *OsERF115/AP2EREBP110* was fused to the 3XHA epitope and transgene expression was controlled by a maize ubiquitin promoter, p*Ubi* (Figure S4a). We generated 35 independent T_0_ transgenic rice plants, analyzed gene expression level by RT-PCR, and selected transgenic lines for further analysis. Figure S4b,c showed qRT-PCR and western blot analysis of four independent homozygous T_2_ lines (OE1, OE2, OE3, and OE4). Expression levels of the modified *OsERF115/AP2EREBP110* transgene (Figure S4b; left panel) and protein (Figure S4c) were constitutively higher in these four OE lines than in WT rice plants. We also monitored expression levels of the endogenous *OsERF115/AP2EREBP110* gene in transgenic plants and the result verified that the endogenous gene was not silenced in the transgenic lines (Figure S4b; right panel). Thus, our results indicate that the *OsERF115/AP2EREBP110* transgene was stably integrated into the transgenic rice genome and was effectively expressed under the control of the *Ubi* promoter.

### 2.4. OsERF115/AP2EREBP110-OE Rice Showed Reduced Sensitivity to ABA

Our results showed that *OsERF115/AP2EREBP110* suppressed the ABA-induced transcriptional activation of the *Rab16A* promoter in rice protoplasts (Figure 2b). Therefore, we investigated the transcript level of *Rab16A* and ABA responses in *OsERF115/AP2EREBP110*-OE rice. qRT-PCR analysis indicated that the *Rab16A* transcript level was highly induced by ABA in WT seedlings whereas the ABA-induced expression of *Rab16A* was significantly suppressed in *OsERF115/AP2EREBP110*-OE rice (Figure 2c).

We evaluated the sensitivity of *OsERF115/AP2EREBP110*-OE seedling growth to exogenous ABA. There was no significant difference in seedling growth of OE and WT plants in the absence of exogenous ABA (Figure 2d). However, in the presence of 3 and 10 µM ABA, the shoot and root growth of OE seedlings was less sensitive to ABA compared to WT seedlings (Figure 2d). Our results indicate that overexpression of *OsERF115/AP2EREBP110* reduced ABA sensitivity in the seedling growth stage and decreased ABA-induced expression of the *Rab16A* gene, suggesting that *OsERF115/AP2EREBP110* could be a negative regulator of the ABA response.

### 2.5. Overexpression of OsERF115/AP2EREBP110 Enhances Thermotolerance of Mature Seeds and Vegetative Stage Plants

**Figure 3:**
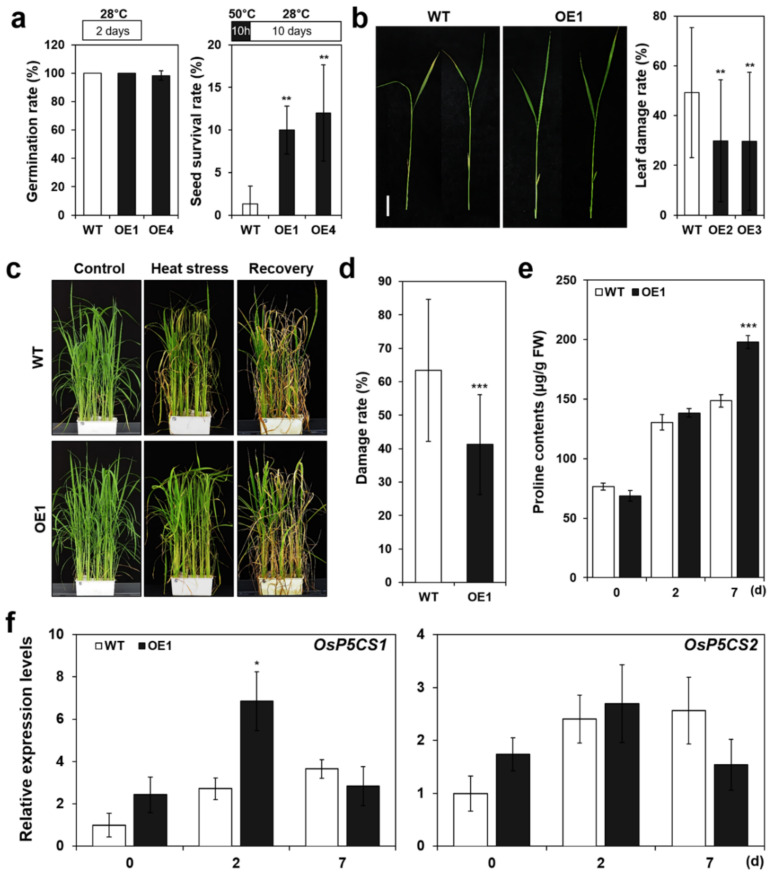
*OsERF115/AP2EREBP110*-OE transgenic rice shows enhanced thermotolerance in mature seeds and vegetative stage plants. (**a**) Seed survival rate of WT and *OsERF115/AP2EREBP110*-OE lines after exposure to heat shock (50 °C). Surface-sterilized and imbibed husked seeds were placed on 1/2 MS medium and incubated for 10 h at 50 °C. After 10-days recovery at 28 °C, number of green seedlings germinated from the seeds were counted. Data represent mean (± SD) from three independent experiments with two biological replicates (*n* = 25). (**b**) Phenotypes of *OsERF115/AP2EREBP110*-OE and WT seedlings exposed to heat stress (37 °C) for 7 days at V2 growth stage. Scale bar: 4 cm. Photographs were taken at 7-days after heat treatment and leaf damage rate of each plant was scored. Data represent the mean (± SD) from three independent experiments with two biological replicates (*n* = 25). One-way ANOVA and Tukey’s HSD test were performed with a comparison of WT plants as controls (**, *p* < 0.01; ***, *p* < 0.001). (**c**–**d**) Phenotypes of WT and *OsERF115/AP2EREBP110*-OE plants exposed to heat stress (42 °C) at V6 growth stage. Plants grown in a soil pot were exposed to heat stress (42 °C) for 7 days and then recovered at 28 °C for 7 days. Photographs were taken at 7-days after recovery (**c**) and damage rate of each plant was scored (**d**). Data represent the mean (± SD) three independent experiments with two biological replicates (*n* = 25). Student’s *t*-test was performed through comparison with rice seeds exposed to 30 °C as controls (***, *p* < 0.001). (**e**) Proline contents of WT and *OsERF115/AP2EREBP110*-OE plants after 2- and 7-days of exposure to heat stress (42 °C). Data represent mean (± SD) from three independent experiments with two biological replicates (*n* = 3). (**f**) Relative expression levels of proline synthesis-related genes (*OsP5CS1* and *OsP6CS2*) in OE and WT plants exposed to 42 °C heat stress for 2 and 7 days. Data represent mean (± SD) from three independent experiments with three biological replicates (*n* = 3). Two-way ANOVA and Fisher’s LSD test were performed by comparing with WT plants as controls (*, *p* < 0.05; ***, *p* < 0.001).

Our data indicate that transcript level of *OsERF115/AP2EREBP110* was elevated by heat stress in both ripening seeds and vegetative tissues (Figure 1). Therefore, it is predicted that *OsERF115/AP2EREBP110* would function in seed germination or seedling growth under high temperature condition. Thermotolerance of *OsERF115/AP2EREBP110*-OE plants was evaluated under the various thermal conditions. First, we scored seed germination rates at different temperature conditions (30 °C, 42 °C, and 50 °C) for 5 days, but could not find significant differences between transgenic and WT mature seeds as shown in Figure 6c in Section 2.8. Second, the transgenic and WT seeds were subjected to heat shock at 50 °C for 10 h and then the survival rate of embryos emerged from the seeds was investigated. Transgenic and WT seeds showed similar germination rates at 28 °C (Figure 3a; left panel). However, after exposing the seeds to heat shock and recovering them for 10 days at 28 °C, the number of germinated seeds of OE plants increased by about 11% compared to WT plants (Figure 3a; right panel). These results suggest that *OsERF115/AP2EREBP* could contribute to protect the seeds from the heat injury.

We further assessed the heat stress responses of the rice plants at growth stages V2 and V6 as described by Counce et al. [51]. The damaged symptom at the leaf tip of OE seedlings was less severe than that of the WT plants under 38 °C heat stress (Figure 3b; left panel). When the severity of the heat stress-induced damage was scored, the damage rate of the WT plants was 21% higher than that of the OE plants (Figure 3b; right panel). In order to confirm whether heat tolerance continued even in the V6 stage, transgenic plants were exposed to 42 °C heat stress for seven days. Before stress treatment, there was no phenotypic difference between the OE and WT plants (Figure 3c). However, under heat stress conditions, chloroplast destruction at the leaf tips of the WT plants appeared earlier than in the OE plants, consistent with the results at the V2 stage (Figure 3b,c). As a result, the damage rate of the WT plants was 22% higher than that of the OE plants (Figure 3d).

To investigate physiological changes associated with the enhanced thermotolerance of *OsERF115/AP2EREBP110*-OE plants, we measured the contents of free proline in OE and WT plants subjected to heat stress. Proline is an important osmoprotectant associated with diverse stress responses in plants. As shown in Figure 3e, free proline content increased by heat treatment of V6 stage plants. After seven days of heat treatment, the proline content in the OE plant was significantly higher than in WT plants (Figure 3e). These results suggest that accumulation of higher proline level in *OsERF115/AP2EREBP110*-OE plants could be a causal reason for the enhanced thermotolerance. The Δ^1^-Pyrroline-5-carboxylate synthetase gene (*P5CS*) encodes the rate-limiting enzyme for proline synthesis. We further analyzed the transcript levels of two rice *P5CS* genes during the heat stress responses of OE and WT plants. As shown in Figure 3f, more rapid and higher accumulation of *OsP5CS1* transcripts were observed in OE plants compared to WT plants when exposed to heat stress. In contrast to *OsP5CS1*, the heat-induced expression of *OsP5CS2* was not significantly different between OE and WT plants (Figure 3f). The increased expression of *OsP5CS1* suggests that *OsP5CS1* is activated by *OsERF115/AP2EREBP110* to affect the free proline content. It is noticeable that expression of the *OsP5CS1* and *OsP5CS2* gene differentially responded to heat stress in *OsERF115/AP2EREBP110*-OE plants (Figure 3f), because the two genes contain different GCC motifs in the promoter region. *OsP5CS1* possesses multiple GCC box and *OsP5CS2* contains a single CE1/GCC box [52].

### 2.6. Phenotyping Water Use Dynamics Using DroughtSpotter Indicates That the OsERF115/AP2EREBP110-OE Transgenic Plants Possess Water Saving Traits under the Heat-Drought Combined Stress

**Figure 4:**
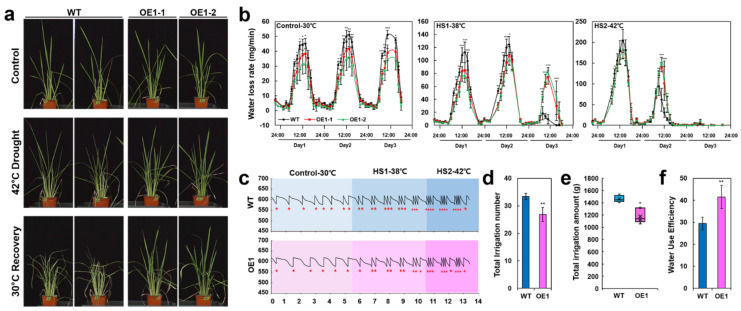
Phenotyping whole plant water use dynamics of *OsERF115/AP2EREBP110*-OE transgenic rice using the DroughtSpotter platform under heat-drought combined stress. (**a**) Photographs of *OsERF115/AP2EREBP110*-OE and WT plants exposed to the combined stress of drought and heat (42 °C) for 7 days and recovered at 30 °C for 7 days. (**b**) Whole plant water loss rates of *OsERF115/AP2EREBP110*-OE and WT plants under NONE mode of DroughtSpotter at three different thermal conditions (Control, HS1, and HS2). Data represent mean (± SD) from two independent experiments with five plants of each line. Two-way ANOVA and Fisher’s LSD test were performed by comparing with WT plants as controls (*, *p* < 0.05; **, *p* < 0.01; ***, *p* < 0.001). Asterisks indicate significant differences in OE1-1 and OE1-2 compared to WT at each time point. (**c**–**e**) Whole plant water use phenotypes of *OsERF115/AP2EREBP110*-OE and WT plants under 7% DYNAMIC mode of DroughtSpotter at three different temperature thermal conditions. (**c**) Representative irrigation graphs from soil-pots of *OsERF115/AP2EREBP110*-OE and WT plants. (**d**) Total number and (**e**) amount of irrigated water input to soil-pots of *OsERF115/AP2EREBP110*-OE and WT plants. Data represent mean (± SD) from two independent experiments with 4 plants of each line. One-way ANOVA was performed by comparing with WT plants as controls (**, *p* < 0.01). (**f**) Whole plant water use efficiency (WP-WUE) of *OsERF115/AP2EREBP110*-OE and WT plants. WP-WUE was calculated by the ratio between the plant area gain and total amount of water input throughout the experimental period. Data represent mean (± SD) from two independent experiments with four plants of each line. Student’s *t*-test was performed by comparing with WT plants as controls (*, *p* < 0.05; **, *p* < 0.01).

High-temperature increases transpiration rate and causes water deficit in diverse plants [53]. We found that *OsERF115/AP2EREBP110*-OE plants showed delayed visual symptoms of water deficit under the combined heat and drought stress conditions compared to WT plants (Figure 4a). This suggests that *OsERF115/AP2EREBP110*-OE plants may possess more positive traits in terms of water saving than wild type plants.

We additionally assessed the water saving traits of individual *OsERF115/AP2EREBP110*-OE plants using an automated irrigation gravitropic platform DroughtSpotter (PHENOSPEX), which is located in a Phytotron in which temperature, humidity, and LED lights were precisely controlled (Figure S5a). The setting conditions of environmental parameters for this experiment are shown in Figure S5. Under the None Mode of DroughtSpotter, the fully watered individual plant was subjected to drought stress by stopping irrigation for three days. Three cycles of drought stress were imposed on each single plant at different thermal conditions (Figure S5b). The maximum temperature of each cycle was 30 °C (Control), 38 °C (HS1), and 42 °C (HS2), respectively. Water loss rates of individual plants sharply increased after onset of light cycle and reached the peak around noon of the day (Figure 4b). As the thermal condition elevated (HS1 and HS2), the water loss rate was markedly increased (Figure 4b). We found that the maximum water loss rate (WR_max_) of the *OsERF115/AP2EREBP110*-OE plant was significantly lower than that of the WT plant during the 30 °C (control) drought cycle. This difference was also evident on day1 of the 38 °C (HS1) drought and thermal cycle (Figure 4b). At day 3 of the HS1 and HS2 drought and thermal cycle, the water loss rate drastically decreased, presumably due to the depletion of soil water content (Figure 4b). At day 3 of HS1 when the wild type plants almost stopped transpiration, the *OsERF115/AP2EREBP110*-OE plants still continued transpiration (Figure 4b).

We further compared the water consumption phenotypes of WT and OE plants using DroughtSpotter in DYNAMIC mode under the same room conditions as Figure S5b. Under this 7% DYNAMIC mode, whenever the weight of the plant pot decreased by 7%, water was replenished to the initial weight of 620 g (Figure 4c). As the thermal condition elevated (HS1 and HS2), the interval time between each irrigation became shorter due to increased water loss rate (Figure 4c). The average irrigation number of the WT and OE plants was 33.4 and 26.8 times, respectively, during the 14-day experimental period (Figure 4d). The total amount of water used for irrigation in the WT plants was 1.24 times more than that of the OE plants (Figure 4e). We estimated the whole-plant water use efficiency (WUE) parameter using the ratio between the plant biomass gain and water irrigated throughout the experimental period as described in the Materials and Methods. As a result, the whole-plant WUE of the *OsERF115/AP2EREBP110*-OE plants was 1.4 times higher than that of the WT plants (Figure 4f). Our data suggest that the OE plants had better water retention ability and used water more efficiently under heat-drought combined stress compared to the WT plants.

### 2.7. OsERF115/AP2EREBP110-Overexpressing Transgenic Plants Keep the Leaf Temperature Lower Than the WT Plants under Heat-Drought Combined Stress

**Figure 5:**
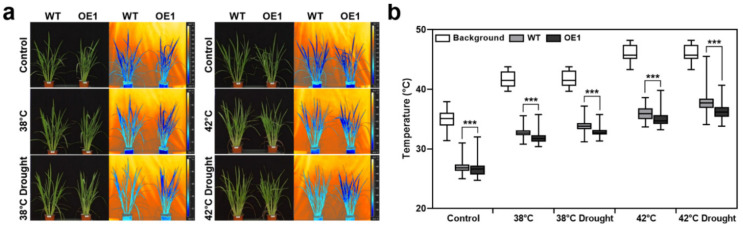
Phenotyping leaf temperature of *OsERF115/AP2EREBP110*-OE transgenic and WT rice plants under heat or heat-drought combined stress. (**a**) Visible and thermal images taken by FLIR P620 camera. Plants at the V6 stage exposed to heat (38 °C or 42 °C) or heat-drought combined stress for 3 days in an environmental controlled Phytotron. All thermal images were taken at the same time when the ambient temperature approached to maximum of the day. (**b**) Average leaf temperature values under heat or heat-drought combined stress. Thermal images were analyzed with FLIR 1.2 SP2 software. Data represent mean (± SD) from 20 pixels from 2~3 leaves of 3 plants of each line with two independent experiments. Two-way ANOVA and Fisher’s LSD test were performed by comparing with WT plants as controls (***, *p* < 0.001).

Leaf temperature reflects the water content and transpiration state of a plant. As the *OsERF115/AP2EREBP110*-OE transgenic plants effectively retained water under the heat-drought combined stress (Figure 4), we implemented the IR thermal imaging method to accurately phenotype leaf temperature changes. Plants were grown in pots in a precisely controlled Phytotron and exposed to three different temperatures and drought stress. Leaf temperature was quantified from the IR thermal images as described in the Materials and Methods (Figure 5a). Before heat stress treatment, leaf temperatures of the fully watered OE and WT rice plants were about 26.88 °C and 26.54 °C, respectively (Figure 5b). When exposed to heat stress of 38 °C and 42 °C, respectively, the average leaf temperatures of the WT plants elevated to 32.68 °C and 35.91 °C, and the OE plants to 31.79 °C and 35.00 °C. When drought stress was additionally imposed with heat stress, the average leaf temperatures were increased to 33.79 °C and 37.71 °C in WT plants and 32.81 °C and 36.16 °C in OE plants under 38 °C and 42 °C thermal conditions, respectively. Our results indicate that rice plants have the ability to keep their leaf temperature 5–7 °C cooler than ambient temperature under the single heat stress or heat-drought combined stress condition. More noticeably, the lower leaf temperature of *OsERF115/AP2EREBP110*-OE plants than WT plants indirectly reflect enhanced water retention capability under heat and drought stress conditions.

### 2.8. Phenotyping Seed Traits of OsERF115/AP2EREBP110-OE Plants

**Figure 6:**
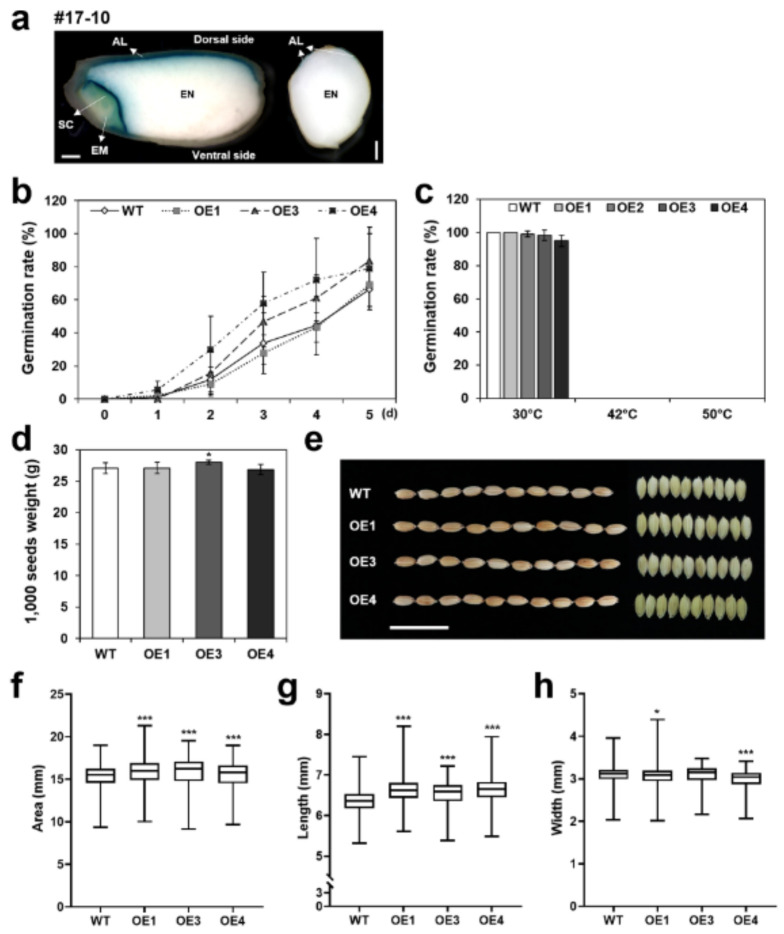
Phenotyping seed dormancy, grain weight, and morphology of *OsERF115/AP2EREBP110*-OE transgenic and WT rice plants. (**a**) Histochemical analysis of GUS expression in the seeds of *pOsERF115/AP2EREBP110*:*GUS* transgenic rice plants. The X-Gluc staining of longitudinal (left) and transverse section (right) of transgenic rice seed are shown. SC, scutellum; EM, embryo; AL, aleurone layer; EN, endosperm; Scale bars: 500 μm. (**b**–**c**) Germination rates of 35 DAH seeds at 30 °C (**b**) or mature seeds at 30 °C, 42 °C, and 50 °C, respectively (**c**). Data represent mean (± SD) three independent experiments with two biological replicates (*n* = 30). (**d**) 1000-grain weights of OE and WT plants. Data represent mean (± SD) from twenty biological replicates (*n* = 100). (**e**–**h**) Image-based analysis of morphological phenotypes of mature seeds. (**e**) Photographs of OE and WT seeds with husk. Scale bar: 2 cm. (**f**) Surface area, (**g**) length, and (**h**) width of seeds with husk measured by high throughput image analysis. Data represent mean (± SD) from 10~30 biological replicates (*n* = 100). One-way ANOVA and Fisher’s LSD test were performed by comparing with WT seeds as controls (*, *p* < 0.05; ***, *p* < 0.001).

Xu et al. previously reported that *OsERF#115* forms a protein complex with the aleurone layer specific OsNF-YB1 and mediates the binding of OsNF-YB1 to GCC boxes of downstream genes during endosperm development [43]. RNAi suppression of OsNF-YB1 retarded grain filling, leading to small grains with chalky endosperms, whereas overexpression of OsNF-YB1 decreased endosperm chalkiness [43]. Public data predict that expression of the *OsERF115/AP2EREBP110* gene is highly specific to reproductive organs and seed endosperm (Figure 1b). To validate the seed-specific expression of *OsERF115/AP2EREBP110*, we generated transgenic rice lines expressing GUS driven by the *OsERF115/AP2EREBP110* promoter (*pOsERF115/AP2EREBP110*:*GUS*) (Figure S4d,e). Our data showed that the *OsERF115/AP2EREBP110* promoter drove expression of GUS in the aleurone layer of mature seeds (Figure 6a). GUS activity was not detected in the endosperm (Figure 6a). This raises the possibility that OsERF115/AP2EREBP110 forms protein complex with OsNF-YB1 in rice aleurone layer cells and plays important roles in regulating endosperm development and grain filling, as suggested by Xu et al. [43].

To assess the potential function of *OsERF115/AP2EREBP110* in seed traits, we phenotyped seed dormancy and morphology of *OsERF115/AP2EREBP110*-OE rice. WT and *OsERF115/AP2EREBP110*-OE rice were planted in a paddy GMO field and seeds were harvested at ripening (35 DAH) and mature stage (65 DAH), respectively. There was no obvious difference in seed germination rates between OE and WT seeds (Figure 6b,c), indicating that seed dormancy was not significantly affected by overexpression of *OsERF115/AP2EREBP110.* The 1000-grain weight of OE plants was also similar to that of WT (Figure 6d). We further conducted image-based high-throughput phenotyping of seed morphology according to the method by Baek et al. [54]. Quantification of area and length of more than thousands of mature single seeds showed a significant increase in grain size of *OsERF115/AP2EREBP110*-OE plants compared with WT plants (Figure 6e–g). At present, it is unknown whether the increased grain size is directly associated with a specific function of *OsERF115/AP2EREBP110* in seed development or an indirect effect from the enhanced stress tolerant performance in the field.

## 3. Discussion

Sustained temperature rises and drought are major environmental factors affecting reduced plant growth and crop yields worldwide. Crops are usually challenged by multiple abiotic stress combinations such as heat, drought, and soil salinity at the same time. It becomes evident that plants develop unique regulatory circuits under the combined abiotic stress conditions [55,56,57,58]. Recent studies have revealed differential plant transcriptomic changes in response to single or combinatory drought, salinity, heat, and cold stresses [59]. Transcription factors, as master regulators of many stress-responsive genes associated with a specific abiotic signaling pathway, are often considered as excellent candidates for crop improvement [60]. Among these, the emerging role of the AP2/EREBP family transcription factors has been noted in a wide range of stress tolerance and hormone signaling in diverse plants [61,62]. To date, numerous studies have focused on the genome-wide identification of stress-responsive AP2/EREBP transcription factors in wild or cultivated plant species toward stress tolerance crop breeding [63,64,65]. Indeed, ectopic expression or gene editing of diverse AP2/EREBP members promote plant tolerance under the cold, salinity or drought stress conditions. For example, over-expression of *OsSTAP1*, AP2/EREBP transcriptional activators, increased salt tolerance of rice [66]. *OsDARP1* confers drought and salinity tolerance of transgenic rice [67]. Overexpression of *OsSta2* enhances the tolerance of transgenic rice plants to salt and osmotic stresses and further leads to increases in grain-filling rate [68]. *OsERF71*, a drought-responsive and root-preferentially expressed gene, confers drought resistance of rice in association with root morphological adaptations [69]. Knock out of *OsEBP89* improves seed germination under submergence and enhances drought tolerance in rice [68,70]. *OsERF101*-overexpressing rice showed higher seed setting rates when subjected to the reproductive-stage drought stress [71]. On the other hand, a few studies have been reported thus far on the function of *AP2/EREBP* genes related to thermotolerance.

Here, we report that overexpression of *OsERF115/AP2EREBP110*, a rice AP2/EREBP Group-IIIc member, improves tolerance to heat-drought combined stress. We identified *OsERF115/AP2EREBP110* as a differentially expressed gene through transcriptomic analysis of ripening rice seeds subjected to heat stress treatment at 25 DAH (Figure 1a). The domain structure of *OsERF115/AP2EREBP110* could not be assigned to any group of Arabidopsis AP2/EREBP family [38]. Based on the conserved motive analysis, Rashid et al. classified *OsERF115/AP2EREBP110* to a small subgroup IIIc of the OsAP2/EREBP family, most of whose function has been unidentified as yet [37]. Besides *OsERF115/AP2EREBP110*, our transcriptome data also indicate that the gene expression of four other IIIc members was induced in the heat-treated ripening seeds (Figure 1a). Recently, a few heat-responsive AP2/EREBP members have been identified by transcriptomic analysis in panicles or vegetative seedlings of Indica rice genotypes with differential heat sensitivity [72,73]. Notably, two Group-IIIc members *OsERF98/AP2EREBP120* and *OsERF109/AP2EREBP63* were identified as ERF transcription factors differentially expressed in panicles of heat tolerant rice cultivars [73]. Additionally, when we monitored the expression profile of these subgroup IIIc members using public data for heat shock transcriptome in rice seedlings, it was noted that gene expression of *OsERF115/AP2/EREBP110* and many other subgroup IIIc members were simultaneously induced by drought, salt, and heat stress (Figure 1c). Taken together, it is suggested that *OsERF115/AP2/EREBP110* and other Group-IIIc members may function as important regulators of heat and drought stress responses in vegetative or reproductive organs.

The *OsERF115/AP2EREBP110*-OE rice exhibited enhanced thermotolerance in seeds and vegetative growth stage plants (Figure 3). In addition, we observed that the whole plant water loss rate of the *OsERF115/AP2EREBP110*-OE rice was significantly lower than that of wild type WT plants when exposed to drought (control) or heat-drought combined stress (HS1 or HS2) conditions on the DroughtSpotter platform (Figure 4b). DroughtSpotter is an automated irrigation gravitropic system. Recently, this system was used to examine the water loss by transpiration of the *lot1* Arabidopsis mutant under drought stress and analyze the WUE of the drought-tolerant *osphyb* rice mutant [21,74]. When we assessed the whole-plant WUE by the ratio between the plant biomass gain (% of initial area) and water irrigated throughout the experimental period, the *OsERF115/AP2EREBP110*-OE rice exhibited higher WUE than WT plants (Figure 4f). Previous studies have shown that drought-tolerant plants have lower water loss and higher WUE in water-constrained environments compared to plants that do not [21,74,75,76,77]. We also showed that the *OsERF115/AP2EREBP110* OE lines exhibited an enhanced drought tolerance phenotype compared to WT plants by the conventional drought stress assay in a greenhouse (Figure S6). These combined results implicate that *OsERF115/AP2EREBP110* functions as a positive regulator of heat and drought stress responses of rice.

The water content of leaf is one of the major physiological indicators that reflects their ability to withstand adversity in stressful environments [78,79]. Heat and drought stress cause decreased water uptake and increased transpiration of plants, significantly impeding efficient water utilization and moisture retention of leaf [7,80]. It has been demonstrated that the leaf-air temperature difference of soybean plants measured through high-resolution thermal IR images under various moisture stresses correlated with the leaf water content [79]. We phenotyped plant temperature by FLIR thermography, and the results indicate that leaf temperature of *OsERF115/AP2EREBP110*-OE plants was lower than WT plants under the heat-drought combined stress conditions (Figure 5). The better ability of *OsERF115/AP2EREBP110*-OE transgenic lines to utilize water efficiently may contribute to conserve water content of the plant and maintain the leaf temperature cooler than the WT plants under the heat-drought combined stress. Indeed, the leaf-air temperature difference (LTD) of the transgenic plants was higher than in WT plants (Figure 5b). Deva et al. reported that heat-resistant common bean (*Phaseolus vulgaris* L.) varieties SAB686 and SEF60 showed enhanced leaf cooling under heat stress than Calima, a heat-sensitive variety, and this difference increased in dry conditions [81]. Swain et al. also found that the leaf water content decreased as the leaf temperature increased in the soybean plant exposed to various water stresses [82]. These studies and our results suggest that over-expression of *OsERF115/AP2EREBP110* contributes to improve heat and drought stress tolerance through enhanced water retention capability and leaf cooling ability.

It is known that ERF proteins regulate biotic and abiotic stress responses by directly or indirectly regulating gene expression through binding with the GCC-box motif or by interacting with other transcription factors [83,84,85,86,87,88]. *Arabidopsis* AtERF1, AtERF2, and AtERF5 act as transcriptional activators by binding to the GCC box in the promoter region of the *Arabidopsis thaliana HOOKLESS1*, which provides a regulatory node in the pathogen and hormonal response pathway, whereas AtERF3 and AtERF4 function as repressors [86]. The tomato ERF protein TSRF1 binds to the *cis*-acting element GCC box in the promoter of a pathogenesis-related gene and positively regulates pathogen resistance in tomatoes and tobacco [87]. This gene also enhanced the expression of *MYB*, *MYC*, and proline synthesis-related genes, which contains the GCC box in their promoter region, leading to improved osmotic and drought tolerance [52]. Similarly, TaERF3 from tomato directly interacts with the GCC box in the promoters of stress-related genes such as *BG3*, *Chit1*, *RAB18*, *LEA3*, *TIP2*, *POX2*, and *GST6*, promoting resistance to salt and drought stress in wheat [83]. In the present study, we showed that *OsERF115/AP2EREBP110* protein targeted to nuclei (Figure 2a), but do not possess transactivation activity in yeast cells and rice protoplasts. Our data are consistent with a previous report by Xu et al. that OsERF#115 binds directly to the GCC box of thee Os07g19790 gene promoter without transactivation activity [43]. Interestingly, we obtained an unexpected result that *OsERF115/AP2EREBP110* suppressed the ABA-mediated transcriptional activation of the *Rab16A* promoter in rice protoplasts (Figure 2b; right panel). *Rab16A* is a representative ABA-responsive gene during seed development and the *pRab16A-*△*660* fragment used in this study contains ABA responsive cis-acting elements (Motif I and Motif II) and a GCC box (Figure S3). Recently, Min et al. showed that *pRab16A-*△*660* is sufficient to monitor ABA signaling in rice protoplasts [49]. Therefore, our result implicates that *OsERF115/AP2EREBP110* may act as a negative regulator of a signaling branch, leading to ABA-dependent transcriptional activation of the *Rab16A* gene. Consistent with this result, we showed that the ABA-inducible expression of *Rab16A* gene was significantly suppressed in the *OsERF115/AP2EREBP110*-OE transgenic lines (Figure 2c). In addition, our result that transgenic lines overexpressing *OsERF115/AP2EREBP110* exhibited reduced sensitivity to ABA in seed germination and post germination growth (Figure 2d) also support the negative role of *OsERF115/AP2EREBP110* in ABA signaling. ABA-mediated stomata closure is a key mechanism to cope with drought stress by decreasing transpiration and water loss. However, we could not find a significant difference in water loss rate from the detached leaves between *OsERF115/AP2EREBP110*-OE transgenic rice and WT plants (Figure S7). Taken together, it is proposed that *OsERF115/AP2EREBP110* positively affects drought and thermotolerance through an ABA-independent regulatory mechanism.

It was noticeable that proline accumulated at a higher level in the *OsERF115/AP2EREBP110*-OE lines than in WT plants when subjected to heat stress (Figure 3e). Proline is an important metabolite associated with adaptive responses against cold, drought, and salt stress. Wang et al. reported that diverse primary and secondary metabolites accumulated at a higher level in transgenic rice overexpressing a drought responsive *ERF* gene, *OsDARP1*. In addition, a recent report revealed that heat stress induces proline accumulation, and the proline overproducing rice by gene editing of *OsProDH* is more resistant to heat stress [89]. Mishura et al. also showed a positive correlation between stress-induced proline content and the multiple stress tolerance of *OsSIZ1* transgenic Arabidopsis [90]. Thus, the leaf proline content could be one causal factor for the enhanced tolerance of *OsERF115/AP2EREBP110*-OE plants under heat and drought stress. We further showed that the transcript level of *OsP5CS1*, a key enzyme for proline synthesis, was higher in the *OsERF115/AP2EREBP110*-OE transgenic rice than in WT plants (Figure 3f), explaining the higher proline content of transgenic rice under the stress condition.

The transcriptional regulatory network of ERF is highly sophisticated. It has been reported that AP2/ERF transcription factors negatively or positively regulate growth and stress response [61]. While many AP2/ERFs are positively involved in the ABA signaling and ABA-mediated stress response, a few studies have reported AP2/ERFs function as a negative regulator of ABA response. The Arabidopsis group X ERF ABR1 (AtERF#111) has a dual function as a repressor of ABA response and activator of wounding or host–pathogen interaction [91,92,93]. The Arabidopsis ORA47 (AtERF18) specifically binds to a novel cis element O-box of ABI2 and inhibits ABA and JA signaling [94]. From our results that *OsERF115/AP2EREBP110* suppressed ABA-inducible *Rab16A*, but activated heat-inducible *P5CS1* gene expression, it is proposed that *OsERF115/AP2EREBP110* may play multiple function as a transcriptional regulator either directly or indirectly.

In conclusion, we demonstrated that *OsERF115/AP2EREBP110* is a novel positive regulator of heat and drought combined stress and a negative regulator of ABA signaling. The enhanced tolerance of *OsERF115/AP2EREBP110*-OE plants is correlated with increased water retention capability. Activation of the *P5CS1* gene, leading to higher proline accumulation, could be a reason for these stress tolerances. Our study provides novel information on the function of the *OsERF115/AP2EREBP110* belonging to the rice Group-IIIc ERF in terms of abiotic stress defense and suggests its potential as candidate gene for genetic engineering approaches to develop heat and drought stress-tolerant crops.

## 4. Materials and Methods

### 4.1. Plant Materials and Growth Conditions

The rice cultivar Dongjin (*Oryza sativa* ssp. *Japonica* cv. Dongjin) was used in this study. Rice seeds were soaked in ipconazole 0.016% for 24 h at 30 °C and rinsed thoroughly with water, placed in a Petri dish (100 × 20 mm^2^) with 15 mL distilled water at 30 °C in dark condition for 4 days, and then transferred to plastic plant trays (50 cells) containing a soil (400 g). After 12 days, the seedlings were transplanted into plastic square pots (60 × 155 × 70 mm^3^) or circular pots (120 × 105 × 84 mm^3^) filled with a rice nursery soil (Seoulbio, Eumseong, Korea) and grown for 3–6 weeks in a greenhouse. Greenhouse was maintained at T_max_ at 30 ± 2 °C during the day and T_min_ at 24 ± 2 °C during the night with relative humidity of 60–70% under long-day conditions (14-h light/10-h dark cycle). For phenotyping water loss dynamics and leaf temperature by DroughtSpotter (PHENOSPEX, Heerlen, The Netherlands) and FLIR P620 infrared camera (FLIR Systems Inc., Wilsonville, OR, USA), respectively, soil pots with a single plant were transferred to a precisely controlled Phytotron (Korea Scientific Technique Industry, Suwon, Korea) operated at 14-h light/10-h dark cycles with 50% relative humidity at 30 °C and were grown for 2–3 days before starting heat-drought experiments.

For seed propagation in the field and the evaluation of seed traits, four-week-old seedlings of transgenic and WT rice plants were transplanted into a paddy GMO field at the National Institute of Agricultural Sciences located at Jeonju, Korea (35°50′ N, 127°4′ E) by 20 × 30 cm intervals with one seedling per hill. When the plants reached maturity, they were harvested and threshed by hands. The average temperature and precipitation conditions during the cultivation period in the paddy GMO field are shown in Table S2. Temperature and precipitation data for the paddy GMO field were obtained from the Korea Meteorological Administration (https://www.weather.go.kr/w/index.do).

### 4.2. Cloning and Expression Analysis of OsERF115/AP2EREBP110 Gene

Rice panicles were harvested at 25 days after heading (DAH) and treated for 5 days at 30 °C and 42 °C, respectively. Total RNAs were extracted from the rice caryopses and transcriptomic profiling was analyzed using the Agilent *O. sativa* GE 180K microarray platform according to the methods described by Chae et al. [95]. *OsERF115/AP2EREBP110* (*LOC_Os08g41030*) was identified as a heat inducible transcription factor from the microarray data. Amino acid sequences of *OsERF115/AP2EREBP110* and other Group-IIIc ERF members were obtained from the Rice Genome Annotation Project Databases (RAP-DB, http://rice.plantbiology.msu.edu/) and conserved motifs were identified using MEME Suite version 5.3.3, a motif-based sequence analysis tool. Tissue-specific expression patterns of the *OsERF115/AP2EREBP110* gene and other Group-IIIc members throughout all developmental phases were monitored by the Rice Expression Profile Database (RiceXPro, http://ricexpro.dna.affrc.go.jp/) and a heat-map was created using Multi Experiment Viewer (MeV; http://www.tm4.org/mev.html). Expression data of *OsERF115/AP2EREBP110* and other Group-IIIc members in rice seedlings exposed to abiotic stresses were obtained from the GEO accession numbers GSE14275 and GSE6901. The stress-inducible expression of the *OsERF115/AP2EREBP110* gene in ripening seeds and vegetative seedlings was verified by qRT-PCR analysis as described in Section 4.6.

The GC content of the *LOC_Os08g41030* gene was very high and it is difficult to isolate the cDNA based on the RT-PCR cloning. A codon-optimized ORF of *LOC_Os08g41030* was synthesized for further functional analysis. The nucleotide sequence of the synthetic ORF showed 79.8% identity with *LOC_Os08g41030* (Figure S2a), but the amino acid sequence was 100% identical (Figure S2b).

### 4.3. Subcellular Localization Analysis in Rice Protoplasts

To analyze subcellular targeting of the *OsERF115/AP2EREBP110* protein, the synthetic ORF of *LOC_Os08g41030* was PCR-amplified and subcloned into the pGEM-GFP-nos vector in frame and the pGEM-GFP-ERF vector was transfected into rice protoplasts according to the method by Bhatnagar et al. [96]. Subcellular localization of the *OsERF115/AP2EREBP110*-GFP fusion protein was observed under a confocal laser scanning microscope TCS SP8 (Leica, Wetzlar, Germany). GFP fluorescent signals were detected with the excitation/emission wavelengths of 488/500 to 550 nm. The red autofluorescence visualizing chlorophylls was captured at emission wavelengths of 680 to 730 nm.

### 4.4. Transcriptional Activation Assay

For transactivation assay in yeast cells, the synthetic ORF of *LOC_Os08g41030* was PCR-amplified, subcloned into the pGBKT7 and fused with the BD domain. The pGBKT7-*OsERF115/AP2EREBP110* construct was transformed into yeast strain AH109 and positive transformants were selected on synthetic dropout (SD) media lacking tryptophan (SD/Trp-). Positive colonies were incubated on the SD/-Trp/-His medium complemented with 0, 0.025, 0.25, and 0.75 mM of 3-AT (3-amino-1, 2, 4-triazole) for three days at 30 °C and transcriptional activation activity was examined.

To assess transactivation activity of *OsERF115/AP2EREBP110* in rice protoplasts, we conducted dual-luciferase assays according to Kim et al. [49]. Two modified *Rab16A* (*LOC_Os11g26790*) promoters fused to firefly luciferase (fLUC) were kindly provided by Dr. Kim BG (National Institute of Agricultural Sciences, Republic of Korea) as reporter vectors. A 548-bp *Rab16A* promoter region (−1512 to −964) containing two DREs (GCCGAC) was fused to the 396-bp (-305 to +91) *Rab16A* promoter region, which harbors ABA responsive cis-elements (Motif I and Motif II), TATA box, and a GCC box (GCCGCC), resulting in a *pRab16A*-△*660:fLUC* construct (same as *pD2-2XDRE:fLUC* in the previous report by Min et al. [50]).

For construction of the effector, the synthetic ORF of *LOC_Os08g41030* was subcloned into the transient expression vector pGEM-UbiHA, which contains the maize ubiquitin promoter and sequence encoding 3XHA tag. The *Renilla* luciferase driven by the UBQ10 promoter (*pAtUBQ-rLUC*) was used as an internal control. Each reporter, effector, and internal control plasmids were co-transfected into rice protoplasts isolated as described above. After transfection, the protoplasts were incubated in W5 medium for 16 h at 28 °C in the presence or absence of 5 μM ABA. Luciferase activity was measured with the Dual-Luciferase Reporter system (Promega, Madison, WI, USA) and GLOMAX 96-microplate luminometer (Promega, Madison, WI, USA) according to the manufacturer’s instructions.

### 4.5. Generation of Transgenic Rice

For generation of *OsERF115/AP2EREBP110*-OE transgenic rice, synthetic ORF of *LOC_Os08g41030* was subcloned into the pGA2897-3XHA vector driven by the maize ubiquitin (*Ubi*) promoter and fused to the sequence encoding 3XHA tag (Figure S4a). The resulting plasmid was transfected into *Agrobacterium* strain LBA4404 and rice transformation was conducted according to Hiei et al. [97]. T_0_ transformants were screened on a medium containing hygromycin (30 µg/µL), transplanted into soil in pots, and grown in a greenhouse. T_1_ or T_2_ transgenic lines overexpressing the synthetic *OsERF115/AP2EREBP110* were selected based on the RT-PCR or qRT-PCR analysis. Expression level of the OsERF115/AP2EREBP110-HA protein in T_2_ homozygous lines were further confirmed by western blot analysis, which we refer to as OE1, OE2, OE3, and OE4.

For generation of *pOsERF115/AP2EREBP110*:GUS transgenic rice, the 5′-upstream 2-kb promoter region of OsERF115/AP2EREBP110 was PCR-amplified and subcloned into the GUS reporter expression vector pBGWFS7 (Figure S4d). T_0_ transformants were selected on a medium containing PPT (DL-Phosphinothricin; 6 mg/mL) and confirmed by PCR analysis (Figure S4e).

### 4.6. Histochemical β-Glucuronidase Assay

For promoter analysis, we used seeds from T_0_ lines of *pOsERF115/AP2EREBP110:GUS* transformants. Twenty three independent T_0_ lines of *pOsERF115/AP2EREBP110:GUS* transgenic rice were generated and grown in a greenhouse. Transgene expression was verified by genomic PCR analysis. For histochemical analysis of GUS expression, more than 15 ripening seeds from four independent T_0_ lines of *pOsERF115/AP2EREBP110*:*GUS* transgenic plants were hand-sectioned with a razor blade. The longitudinal and transverse sections of seeds were incubated at 37 °C in X-gluc reaction buffer (50 mM sodium phosphate buffer, pH 7.0, 0.1% Triton X-100, 1 mM X-gluc, 0.5 mM ferrocyanide, 0.5 mM ferricyanide, and 10 mM EDTA) for 12 h. The GUS-stained seeds were fixed with 70% ethanol and photographed using a Leica S6D stereoscopic microscope (Leica Microsystems, Wetzlar, Germany). Seeds from three independent T_0_ transgenic lines were assayed.

### 4.7. Quantitative Real-Time RT-PCR and Western Blot Analysis

Total RNA was isolated from 7-days rice seedlings using an Inclone^TM^ RNA Mini Extraction Kit (Inclone Biotech, Yongin, Republic of Korea) according to the manufacturer’s instructions. cDNA synthesis was performed with Maxime RT-PCR PreMix (INtRON Biotechnology, Seongnam, Republic of Korea). Real-time PCR analysis was conducted using an AccuPower^®^ 2X Greenstar qPCR Master Mix (BIONEER, Daejeon, Republic of Korea) and 7500 Real Time PCR System (Applied Biosystems, Foster City, CA, USA) according to the manufacturer’s instructions. The mRNA relative quantification was calculated using the 2^−∆∆Ct^ method [98]. The rice ubiquitin 5 (*UBQ5*) and tubuline (*Tub*) gene was used as the internal control in seeds and seedlings, respectively. The primer information is listed in Table S1.

For western blot analysis, total protein was extracted from a single rice seedling (40 mg) using a buffer containing 0.05 M Tris-HCl (pH 7.4), 0.2% SDS, 5% glycerol, 1.5% TritonX-100, 1% β-mercaptoethanol, 1 mM EDTA, 1 mM dithiothreitol, and protease inhibitor cocktail. The protein samples (30 μg) were loaded on 10% SDS-PAGE gels, blotted onto a PVDF membrane (Atto Corp., Tokyo, Japan), and immunoblotted with anti-HA antibody (Santa Cruz Biotechnology, Santa Cruz, CA, USA; cat. no. sc-7392). As an internal control, actin protein level was determined with polyclonal anti-Actin antibody (Agrisera, Vännäs, Sweden; cat. no. AS132640). For detection of the immunoreactive proteins, SuperSignal^TM^ West Femto Maximum Sensitivity Substrate (Thermo Scientific, Waltham, MA, USA) and the Fusion SL Gel Detection System (Vilber Lourmat, Marne-la-Vallée, France) were used.

### 4.8. ABA Sensitivity Assay

To test ABA sensitivity at the seed germination and post-germination growth stage, the husked rice seeds were surface-sterilized and germinated on 1/2 MS medium containing 3 or 10 μM ABA, respectively, at 28 °C under the 14-h light/10-h dark cycle. The shoot and root length of each seedling were measured after seven days of ABA treatment.

### 4.9. Thermotolerance Assay

To evaluate seed germination at high-temperature condition, surface-sterilized seeds were placed in a Petri dish lined with wet filter paper and allowed to germinate for five days under the dark at 30 °C, 42 °C, or 50 °C, respectively. Germination rate was counted as the rate of radicle emergence and sustained growth. Duplicate sets for 30 seeds per Petri dish were counted.

Seed survival rate after heat shock was determined according to the method by Guo et al. with modification [99]. Surface-sterilized husked seeds were placed on 1/2 MS agar medium and allowed to imbibe at 10 °C overnight in the dark. After heat stress treatment for 10 h at 50 °C under the dark, seeds were recovered in a growth room at 16-h light/8-h dark cycles (28 °C) for 10 days. The number of seedlings germinated from the heat stressed seeds was counted as a seed survival rate.

To estimate thermotolerance at vegetative V2 and V6 growth stages, seedlings were either grown on the 1/2 MS agar medium in the growth room or on the soil in a greenhouse at 16-h light/8-h dark cycles (28 °C) for 1–2 weeks. High-temperature stress was imposed at either 37 °C or 42 °C for seven days in an incubator under the continuous light and 14-h light/10-h dark cycles, respectively. After recovery, the number of damaged L1, L2, and L3 leaves of each seedling was counted according to the appearance of visual symptoms such as leaf wilting and yellowing at the tip of the leaf. Seedling survival rate was determined as the number of green seedlings.

### 4.10. Measurement of Proline Content

For analysis of free proline content changes after heat stress, the L4 and L5 leaves of *OsERF115/AP2EREBP110*-OE transgenic and WT lines were sampled before and after 42 °C heat stress treatment for two and seven days. The 0.1 g of sampled leaves were ground, then 0.6 mL 80% (*v*/*v*) ethanol was added, and mixed for 2 min. The mixture was centrifuged at 13,000 rpm for 5 min and the supernatants were collected. The pellet was re-extracted with 0.4 mL of 80% ethanol, then all supernatants were combined. The free proline content dissolved in ethanol was measured according to the method by Park et al. [100].

### 4.11. Leaf Water Loss Assay

For leaf water loss assay, two uppermost leaves of V6 stage rice plants were cut and dried in the air. After exposure for 0, 10, 30, 45, 60, 120, 180, 240, 300, 360, 420, and 480 min, the weight of leaves was measured. Water loss rate was calculated as the percentage of fresh weight after air exposure to fresh weight of the initial tissue.

### 4.12. Experimental Setup for DroughtSpotter under the Heat-Drought Combined Stress

Experiments using DroughtSpotter (PHENOSPEX, Heerlen, The Netherlands) was conducted in a precisely controlled Phytotron (Korea Scientific Technique Industry, Suwon, Republic of Korea), operated at 14-h light/10-h dark cycles with 50% relative humidity under the three different thermal conditions where the maximum temperature was 30 °C (Control), 38 °C (HS1), and 42 °C (HS2), respectively. Setting conditions for temperature and LED light of a day are shown in Figure S5b. Ambient condition of the Phytotron was monitored in real time with environmental sensors during the experimental periods (Figure S5c).

Our DroughtSpotter system consists of 48 load cells, each automatically weighing every one minute. A single plant was grown in a pot (120 × 105 × 84 mm^3^) for 3–4 weeks and each pot immersed on a plastic tray loaded on a DroughtSpotter cell. The timing and amount of irrigation were precisely controlled at each loading cell. Before starting the experiment, the weight of each pot was equalized to 620 g with water-saturated soil. Each pot irrigated between 20:00 and 20:30 of a day. To impose drought-heat combined stress, DroughtSpotter was operated with two irrigation systems, NONE mode and DYNAMIC mode under the three different thermal conditions, respectively. At the NONE mode, drought was imposed by stopping water supply for three days and the weight of each pot was automatically measured every 1 min. At the end of three days-drought stress, each pot was replenished with water for two days to restore the turgor of the plants, and then drought stress was imposed again under elevated thermal conditions (HS1 and HS2). An empty soil pot without plants was used as a reference to estimate the effect of the ambient condition on the water evaporation from the soil. Reading data were combined over 90 min periods and the water loss rate (mg/min) of the individual plant was calculated according to the formula below. Average water loss rate of five individual plants of wild type and *OsERF115/AP2EREBP110*-OE lines was calculated in biological repeat experiments.

Whole plant water loss rate (mg/min) = [(Plant pot weight loss every 90 min) − (Empty pot weight loss every 90 min)]/90.

At the DYNAMIC mode, each pot was automatically irrigated to adjust the final weight to 620 g whenever the plant pot weight was reduced by 7% after the start of the experiment. The DYNAMIC mode was operated for five days under three different thermal conditions, respectively, the same as in the NONE mode. The number and amount of irrigated water for each pot were measured while the plants were growing under stress conditions. Before and after the experiments, the RGB image of each plant was obtained using a digital camera and the plant area was estimated using the ImageJ program. The whole-plant water use efficiency (WP-WUE) was calculated by the ratio between the plant area gain and water irrigated throughout the experimental period. Average WUE of four individual plants of wild type and *OsERF115/AP2EREBP110*-OE lines was calculated in biological repeat experiments.

Whole plant WUE = Plant area gain (% of initial area)/total amount of irrigated water.

### 4.13. Phenotyping Leaf Temperature under Heat-Drought Combined Stress Using Infrared Thermal Imaging

A FLIR P620 infrared camera (FLIR Systems Inc., Wilsonville, OR, USA) was used to take thermal images under the heat-drought combined stress condition. A single plant was grown in a single pot for 4–5 weeks and drought stress was imposed at three different thermal conditions in a phytotron as described in Section 4.12. After heat-drought stress treatment, re-watering and recovery of the plants were performed at 30 °C. All thermal images were taken from 13:00 to 15:00 PM when the ambient temperature approached the maximum of the day. A black cloth was set up behind the rice plants when record thermal images. The pixel resolution and wavelength band of infrared thermal camera were 640 × 480 pixels and 7–14 μm, respectively, and the distance between the plant and the sensor was 1.5 m. Images were analyzed using FLIR 1.2 SP2 software. The average leaf surface temperature was measured from the randomly selected pixels along with a single leaf of each plant’s thermal image. More than 20 pixels from the 2–3 leaves of each plant with three biological repeats were used to calculate the leaf temperature.

### 4.14. Phenotyping Seed Traits

Fully filled grains were used to measure grain weight and size of the WT and OE lines. Grain weight was calculated by 100 grains and converted to 1000-grain weight. Grain size was estimated from the projected area, major (length) and minor (width) parameters by high-throughput image-based analysis as described by Baek et al. [54]. A total of 1000–3000 grains of each line were used to calculate a single grain size.

## Data Availability

The data presented in this study are available in article or Supplementary Materials here.

## Supplementary Materials

The following are available online at https://www.mdpi.com/article/10.3390/ijms22137181/s1. List of Supplementary Data: Figure S1. Locations and amino acid sequences of five conserved motifs among the *OsAP2/EREBP* Group-IIIc members determined by MEME Suite version 5.3.3, Figure S2. (a) Nucleotide and (b) amino acid sequence alignments of wild type and codon-optimized synthetic ORF of *OsERF115/AP2EREBP110* (*LOC_Os08g41030*) gene, Figure S3. Schematic diagrams of wild type (*pRab16A*) and modified (*pRab16A-Δ660*) promoter of *Rab16A* gene, Figure S4. Expression analysis of *OsERF115/AP2EREBP110*-OE and *pOsERF115/AP2EREBP110*:*GUS* transgenic rice lines, Figure S5. Schematic flow chart of phenotyping whole plant water use dynamics using the DroughtSpotter platform in an environmentally controlled Phytotron, Figure S6. Phenotypes of WT and *OsERF115/AP2EREBP110*-OE plants exposed to drought stress at the V6 growth stage, Figure S7. Water loss rates in leaves of *OsERF115/AP2EREBP110*-OE transgenic and WT rice plants, Table S1. Primer sequences used in this study, Table S2. Weather conditions during the cultivation periods (June–October) of the paddy GMO field in 2020.
