# Supplementary material for: The OsERF115/AP2EREBP110 Transcription Factor Is Involved in the Multiple Stress Tolerance to Heat and Drought in Rice Plants
Source: Int J Mol Sci. 2021 Jul 2;22(13):7181. doi: 10.3390/ijms22137181 (PMC8269390; doi:10.3390/ijms22137181)
Supplement: Supplementary file 1 [file ijms-22-07181-s001.zip › ijms-1239275-SI.pdf]

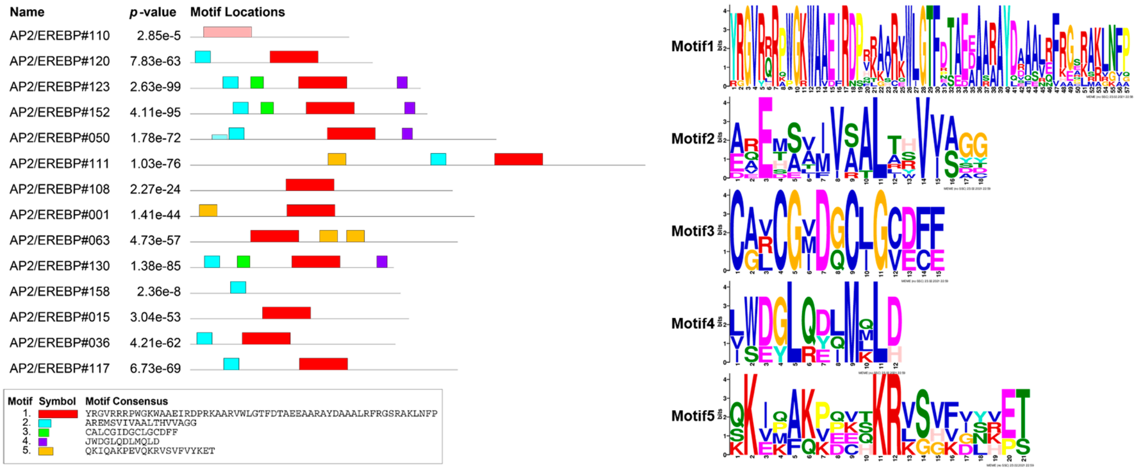

Figure S1. Locations and amino acid sequences of five conserved motives among *OsAP2/EREBP* Group-IIIc members determined by MEME Suite version 5.3.3. Each block shows the position and strength of a motif site. The blocks in solid color are motif sites predicted by MEME. The Motif 1 (AP2/ERFBP domain; red block) of *OsERF115/AP2EREBP110* was detected by MEME plus scanning algorithm based on the de-novo motif identification (solid red color) and indicated as transparent red color block.

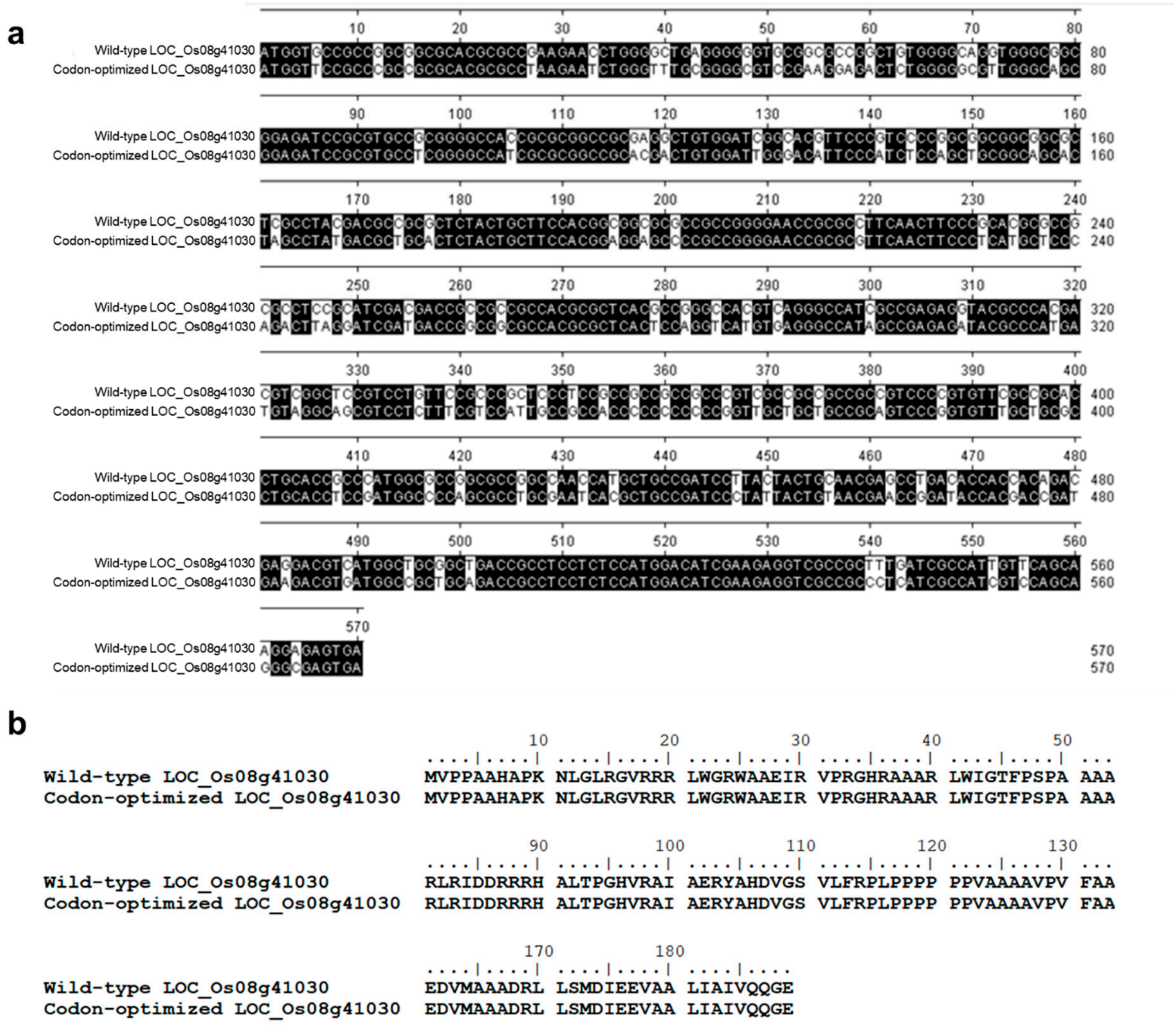

Figure S2. (a) Nucleotide and (b) amino acid sequence alignments of wild type and codon-optimized synthetic ORF of *OsERF115/AP2EREBP110* (*LOC_Os08g41030*) gene.

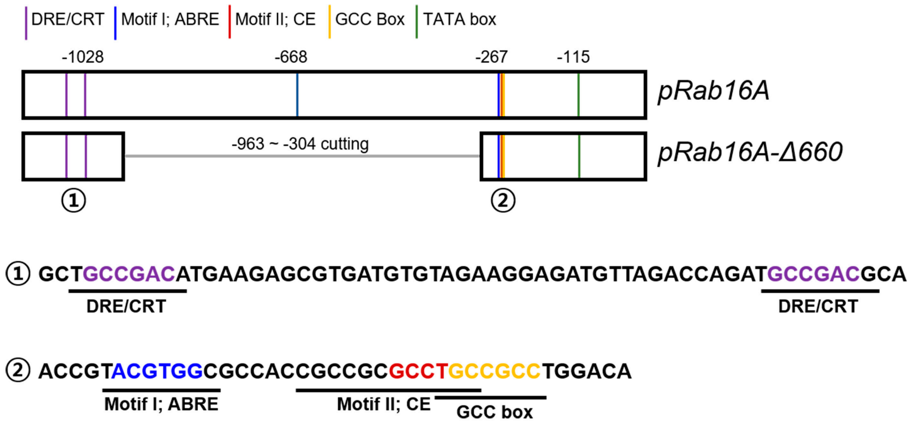

Figure S3. Schematic diagrams of wild type (*pRab16A*) and modified (*pRab16A-Δ660*) promoter of *Rab16A* gene. Positions and nucleotide sequences of cis-acting elements for DREB (DRE/CRT) and ERF (GCC box) binding or ABA responsive elements (Motif I and Motif II) were indicated.

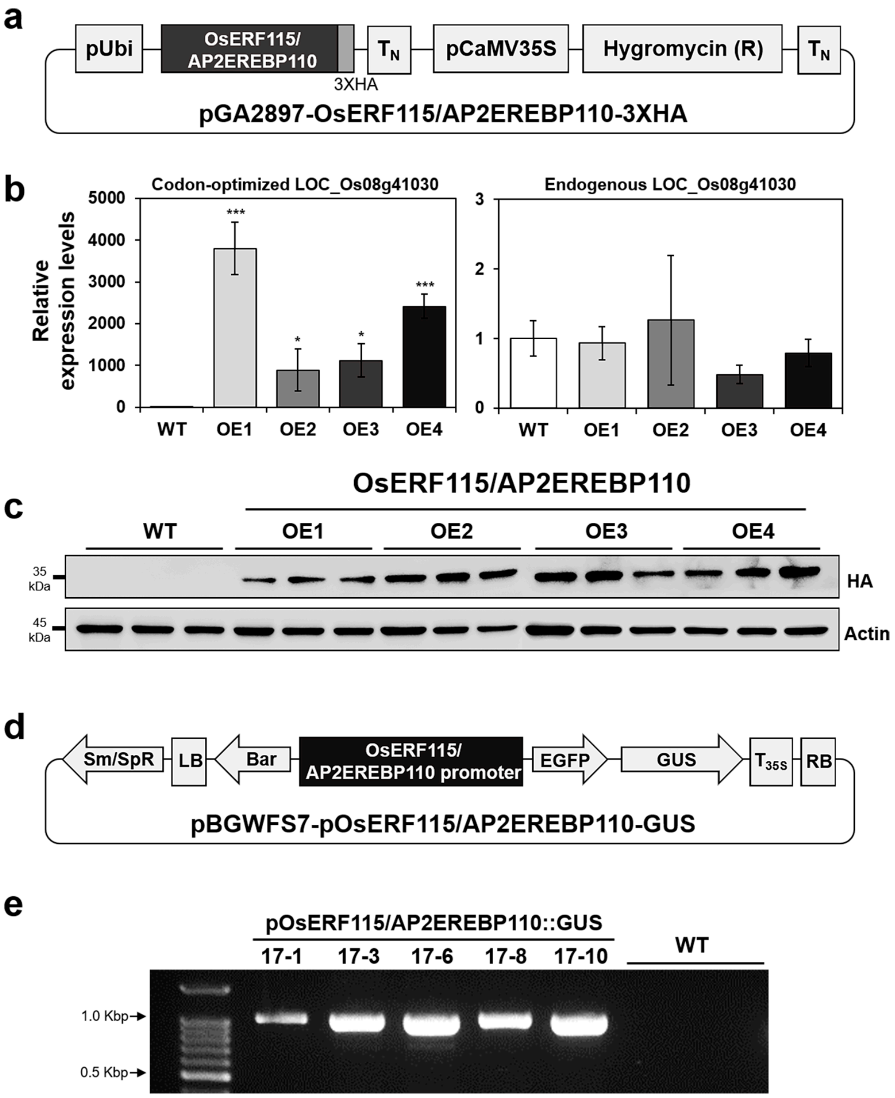

Figure S4. Expression analysis of *OsERF115/AP2EREBP110*-OE and *pOsERF115/AP2EREBP110::GUS* transgenic rice lines. (**a**) Schematic diagram of vector map used for *OsERF115/AP2EREBP110*-OE transgenic rice transformation. (**b**) qRT-PCR analysis of transcript level of codon-optimized and endogenous (wild-type) *OsERF115/AP2EREBP110* gene in WT and OE lines. To discriminate expression of transgene and endogenous *OsERF115/AP2EREBP110* gene, primer sets specific to codon-optimized or wild-type sequences were used for qRT-PCR (OsERFM-F/R and OsERF-F2/R2 in Table S2). Data represent mean ± standard deviation (SD) from three independent experiments with three biological replicates (n=3). One-way ANOVA and Fisher's LSD test were performed comparing with untreated control (*, P < 0.05; ***, P < 0.001). (**c**) Western blot analysis of OsERF115/AP2EREBP110-HA protein level in WT and OE lines. Protein extracts from rice seedlings were mixed with 5X SDS-PAGE loading buffer, resolved in SDS-PAGE, and immunoblotted with anti-HA antibody. As an internal control, actin protein level was determined with anti-Actin antibody. (**d**) Schematic diagram of vector map used for *pOsERF115/AP2EREBP110:GUS* transgenic rice transformation. (**e**) Genomic PCR confirmation of *pOsERF115/AP2EREBP110:GUS* transgenic plants

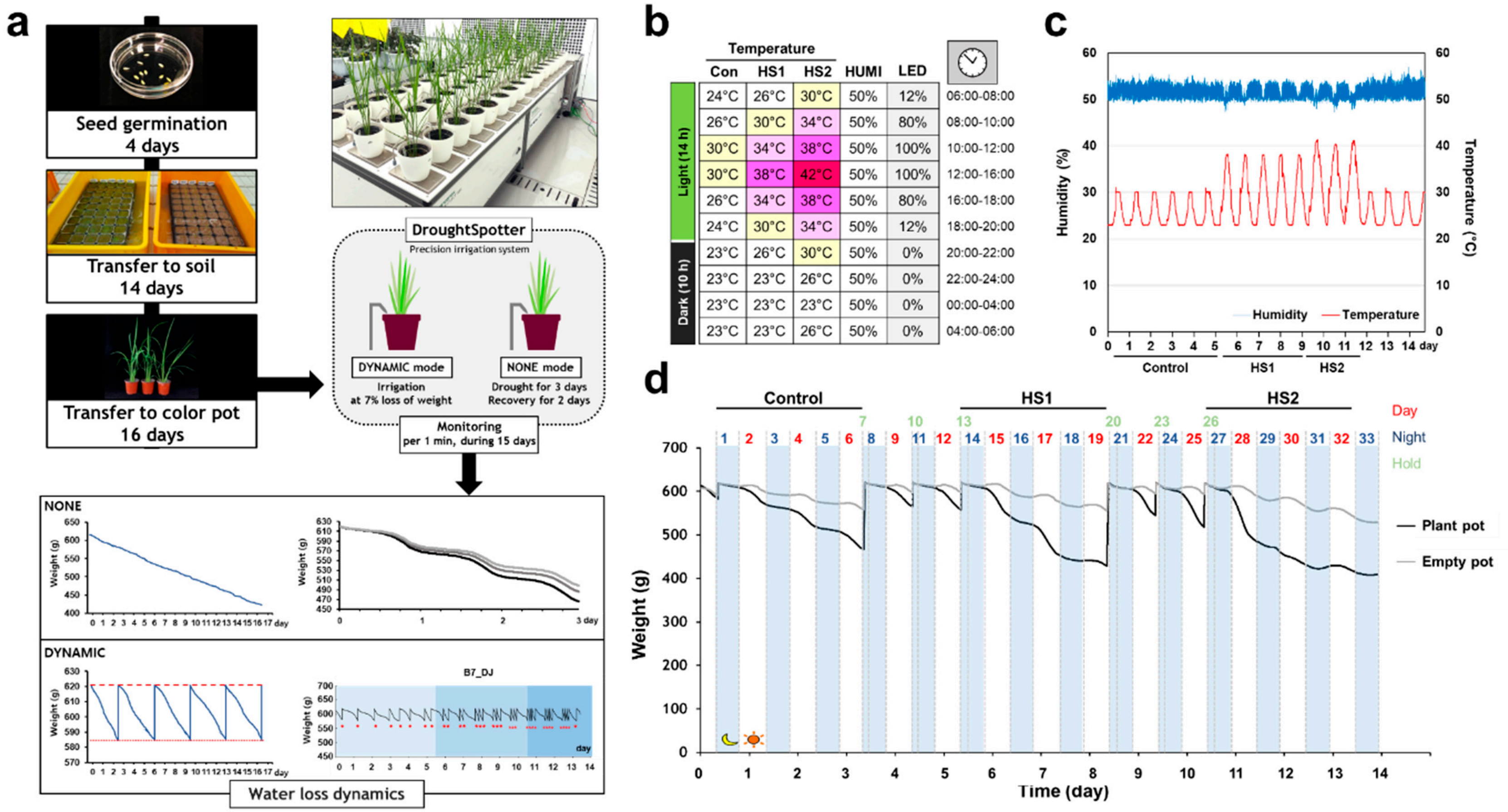

Figure S5. (**a**) Schematic flow chart of phenotyping whole plant water use dynamics using DroughtSpotter platform in environmental controlled Phytotron. (**b**) Daily settings of temperature, relative humidity, and LED light under three different thermal conditions (Control, HS1 and HS2). (**c**) Real-time monitoring of ambient temperature (red line) and humidity (blue line) during the experiment. (**d**) Representative weight loss curves from soil pots with or without single plant at the NONE mode of DroughtSpotter platform under three different thermal conditions.

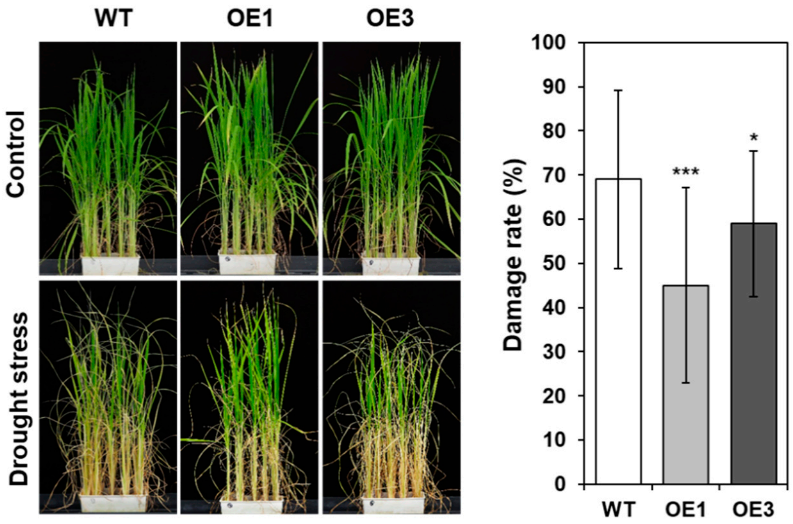

Figure S6. Phenotypes of WT and *OsERF115*/*AP2EREBP110*-OE plants exposed to drought stress at V6 growth stage. Twenty plants were planted in a square soil pot and grown until V6 stage in a greenhouse. Drought stress was imposed for 2 days and then plants were recovered for 7 days under the greenhouse condition. Photographs were taken at 7-days after recovery (left panel) and damage rate of each plant was scored (right panel). Data represent the mean (± SD) from three independent experiments with two biological replicates (n=20). One-way ANOVA and Fisher's LSD test were performed comparing with WT plants as controls (*, $P < 0.05$; ***, $P < 0.001$).

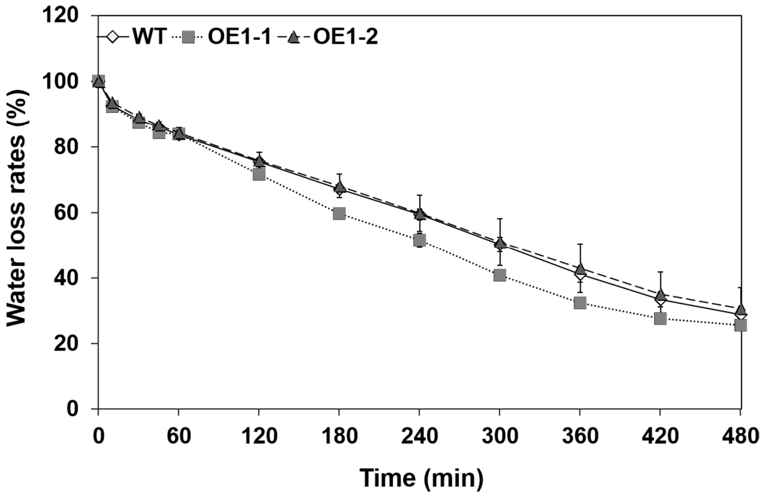

Figure S7. Water loss rates in leaves of *OsERF115/AP2EREBP110*-OE transgenic and WT rice plants. Plants were grown in a greenhouse until V6 stages. Two leaves from the top of the main tiller were cut and air-dried at 28°C. Data represent mean (± SD) from three biological repeats with three replicates of each line.

Table S1. Primer sequences used in this study.

Oligo name	Oligonucleotide sequences (5´-3´)
Tub-F	GAGTACCCTGCCGCATGAT
Tub-R	GTGGTCAGCTTGAGAGTCCT
UBQ5-F	AGAAGCGCAAGAAGAAGACG
UBQ5-R	GCGTCGTCCACCTTGTAGA
OsERF-F1	TGATCGCCATTGTTCAGCAAG
OsERF-R1	GGTGCATGACCAAGTACAGA
OsERFM-F	CTATTACTGTAACGAACCGGATACCA
OsERFM-R	GCGACCTCTTCGATGTCCAT
OsERF-F2	CAAACAGATGCGCCAGGTT
OsERF-R2	TGCACGCATGCAGGAGTAAC
OsRab16A-F	CACACCACAGCAAGAGCTAAGTG
OsRab16A-R	TGGTGCTCCATCCTGCTTAAG
OsP5CS1-F	CCCGTCCCGGAGCTTCGTGAG
OsP5CS1-R	CCTAAGTCGCTGTCGCCCCAC
OsP5CS2-F	GCTGCCGTCGGTCAGAGTG
OsP5CS2-R	CTCGTATGGTTGCCTCCTGGT

Table S2. Weather conditions during the cultivation periods (June–October) of the paddy GMO field in 2020.

	June		July		August		September		October	
	2020	Mean value	2020	Mean value	2020	Mean value	2020	Mean value	2020	Mean value
Mean Temp. (°C)	23.4	22.8	23.5	25.6	27.7	27.7	21.3	21.9	14.9	15.1
Max. Temp. (°C)	28.7	28.3	27.3	29.9	32	32.3	26.5	26.8	20.9	20.7
Min. Temp. (°C)	19.5	18.6	20.8	22.3	24.6	24.2	17.6	18	9.8	10.4
Total Precip. (mm)	187.5	137.9	644.7	330	471.5	319.3	135.2	143.9	4.2	66.8

* Mean Temp., mean temperature; Max. Temp., maximum temperature; Min. Temp., minimum temperature; Total Prepcip., total precipitation; Mean value, Mean value from 2018 to 2020
